# Highly Contaminated Marine Sediments Can Host Rare Bacterial Taxa Potentially Useful for Bioremediation

**DOI:** 10.3389/fmicb.2021.584850

**Published:** 2021-03-01

**Authors:** Filippo Dell’Anno, Eugenio Rastelli, Michael Tangherlini, Cinzia Corinaldesi, Clementina Sansone, Christophe Brunet, Sergio Balzano, Adrianna Ianora, Luigi Musco, Maria Rita Montereali, Antonio Dell’Anno

**Affiliations:** ^1^Stazione Zoologica Anton Dohrn, Naples, Italy; ^2^Department of Materials, Environmental Sciences and Urban Planning, Polytechnic University of Marche, Ancona, Italy; ^3^Laboratory of Marine Biology and Zoology, DiSTeBA, University of Salento, Lecce, Italy; ^4^ENEA - Agenzia per le Nuove Tecnologie, l'Energia e lo Sviluppo Economico Sostenibile, Rome, Italy; ^5^Department of Life and Environmental Sciences, Polytechnic University of Marche, Ancona, Italy

**Keywords:** marine sediments, microbial diversity, polycyclic aromatic hydrocarbons, heavy metal resistance, bioremediation

## Abstract

Coastal areas impacted by high anthropogenic pressures typically display sediment contamination by polycyclic aromatic hydrocarbons (PAHs) and heavy metals (HMs). Microbial-based bioremediation represents a promising strategy for sediment reclamation, yet it frequently fails due to poor knowledge of the diversity and dynamics of the autochthonous microbial assemblages and to the inhibition of the target microbes in the contaminated matrix. In the present study, we used an integrated approach including a detailed environmental characterization, high-throughput sequencing and culturing to identify autochthonous bacteria with bioremediation potential in the sediments of Bagnoli-Coroglio (Gulf of Naples, Mediterranean Sea), a coastal area highly contaminated by PAHs, aliphatic hydrocarbons and HMs. The analysis of the benthic prokaryotic diversity showed that the distribution of the dominant taxon (Gammaproteobacteria) was mainly influenced by PAHs, As, and Cd concentrations. The other abundant taxa (including Alphaproteobacteria, Deltaproteobacteria, Bacteroidetes, Acidobacteria, Actinobacteria, NB1-j, Desulfobacterota, and Myxococcota) were mainly driven by sediment grain size and by Cu and Cr concentrations, while the rare taxa (i.e., each contributing <1%) by As and aliphatic hydrocarbons concentrations and by sediment redox potential. These results suggest a differential response of bacterial taxa to environmental features and chemical contamination and those different bacterial groups may be inhibited or promoted by different contaminants. This hypothesis was confirmed by culturing and isolating 80 bacterial strains using media highly enriched in PAHs, only nine of which were contextually resistant to high HM concentrations. Such resistant isolates represented novel Gammaproteobacteria strains affiliated to *Vibrio*, *Pseudoalteromonas*, and *Agarivorans*, which were only scarcely represented in their original assemblages. These findings suggest that rare but culturable bacterial strains resistant/tolerant to high levels of mixed contaminants can be promising candidates useful for the reclamation by bioaugmentation strategies of marine sediments that are highly contaminated with PAHs and HMs.

## Introduction

Bioremediation of marine sediments contaminated by polycyclic aromatic hydrocarbons (PAHs) and heavy metals (HMs) is a major priority for coastal areas impacted by industrial activities, wastewater discharge, and other anthropic pressures (Burgos-Núñez et al., 2017; Jupp et al., 2017; Mali et al., 2017; Monaco et al., 2017; Dell’Anno et al., 2018). Common microbial-based bioremediation strategies include the addition of specific compounds to stimulate autochthonous bacteria (biostimulation) and/or the addition of specific bacterial strains, which display useful biodegradation/detoxification capacity (bioaugmentation; Beolchini et al., 2007, 2009, 2011; Das and Chandran, 2011; Adams et al., 2015; Fodelianakis et al., 2015). For effective bioremediation, microbes should be (i) abundant and metabolically active in the natural system, (ii) resistant to mixed contaminations, (iii) easy to isolate/grow, and/or (iv) responsive to biostimulation (Mohajeri et al., 2017; Dell’Anno et al., 2018; Mahjoubi et al., 2018; Singh et al., 2020). However, frequent failures are observed in biostimulation due to unsuccessful increase in the abundance of target microbes, as well as in bioaugmentation due to the low fitness of the lab-grown microbes once they are released in the contaminated environment (Goldstein et al., 1985; Stephenson and Stephenson, 1992; Bouchez et al., 2000; Vogel and Walter, 2001; Wagner-Döbler, 2003).

Improving our knowledge on the diversity and dynamics of natural microbial assemblages in the target benthic systems can provide useful information for optimizing biostimulation approaches and thus bioremediation performance (Thompson et al., 2005; Carter et al., 2006; Tyagi et al., 2011; Dell’Anno et al., 2012; Rocchetti et al., 2012; Atashgahi et al., 2017). At the same time, information on the sensitivity of the lab-grown microbial strains selected for the bioremediation of a specific contaminant toward other types of contaminants co-present in the target sediment is required for improving the effectiveness of bioaugmentation approaches (Wild et al., 1991; Thompson et al., 2005; Pepi et al., 2009; Tyagi et al., 2011; Dueholm et al., 2015).

Thus, a robust approach aimed to minimize the risks of failure in bioremediation of marine sediments based on biostimulation should assess the actual presence and relative abundance of useful and responsive microbes in the natural system (Thompson et al., 2005; Alisi et al., 2009). Moreover, it should assess if the microbial strains used for bioaugmentation are tolerant toward other co-occurring contaminants (Duxbury 1985; Insam et al., 1996; Dell’Anno et al., 2003; Nwuche and Ugoji, 2008; Alisi et al., 2009).

Bagnoli-Coroglio, in the Gulf of Naples (Mediterranean Sea), is a typical example of a coastal area highly contaminated by metals and hydrocarbons, which have been released for decades by industrial activities and stopped at the beginning of 1990s (Romano et al., 2004, 2008, 2018; Arienzo et al., 2017; Trifuoggi et al., 2017; Armiento et al., 2020; Morroni et al., 2020). However, other sources of contamination, including urban and sewage discharge, continue to affect the coastal area, which is thus exposed to multiple forms of human pressure deriving from both past and recent activities (Bertocci et al., 2019). Recent data show that the concentrations and distribution of contaminants in the sediments have remained quite constant over the last 15 years, even if organic and inorganic contaminants have been spreading especially northward along the coast and toward the open sea in the area in front of the piers of the dismissed ILVA steel plant (Armiento et al., 2020). Despite the urgent need for remediation and management actions in the coastal area of Bagnoli-Coroglio, no active remediation of the contaminated marine sediments has been attempted to date (Morroni et al., 2020).

In the present study, we used an integrated approach including a detailed environmental characterization, next-generation sequencing and culturing to identify autochthonous bacteria with bioremediation potential in the sediments of the Bagnoli-Coroglio coastal area.

## Materials and Methods

### Sampling Area and Sediment Processing

**Figure 1:**
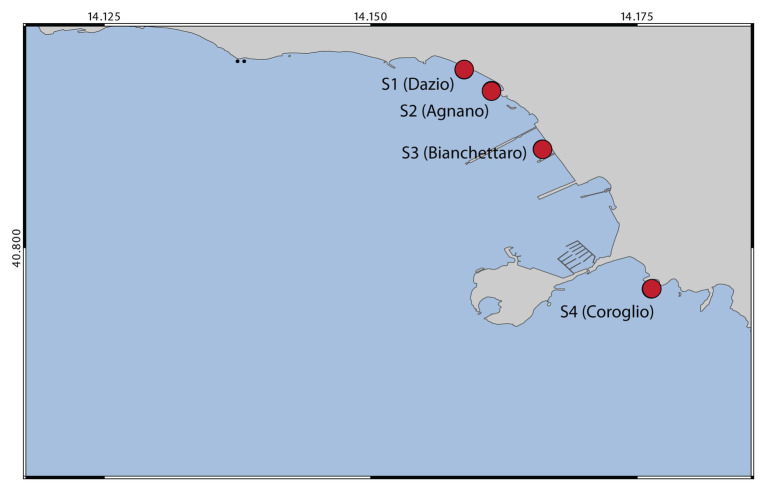
Map showing the location of the four sampling sites from which sediment samples were collected.

Sediment samples were collected in April 2017 at four sites, named Dazio, Agnano, Bianchettaro, and Coroglio (hereafter defined sites S1, S2, S3, and S4, respectively) located along the coast of Bagnoli-Coroglio (Naples, Tyrrhenian Sea; Figure 1) and representative of the multifaceted conditions that characterize the study area, since these sites are affected by historical industrial contamination as well as by recent contamination deriving from the surrounding heavily urbanized area. At each site, surface sediment samples were collected in triplicates using sterile Plexiglas cores, and sediment pH and redox potential (Eh) were immediately recorded in the fresh samples using a portable pH/EC/TDS meter HI9813-5 (Hanna Instruments). All sediments were collected on the same sampling date and at similar depths and temperature (approximately 2 m depth and 18.5°C). Fresh sediment sub-aliquots were used for bacterial isolation and identification, while other sediment aliquots stored at −20°C were used for the determination of PAH, HM, and aliphatic hydrocarbon concentrations, grain size and quantity and biochemical composition of the organic matter, and for prokaryotic abundance and diversity.

### PAHs, Aliphatic Hydrocarbons (C > 12), and HMs Concentrations

Polycyclic aromatic hydrocarbons once extracted from the sediment samples according to EPA 3545A procedure were then analyzed by gas chromatography-mass spectrometry (GC-MS; EPA 8270D). The total PAH concentrations were obtained summing those of the 16 congeners quantified (naphthalene, acenaphthene, fluorene, acenaphthylene, phenanthrene, anthracene, fluoranthene, pyrene, benzo[a]anthracene, chrysene, benzo[b]fluoranthene, benzo[k]fluoranthene, benzo[a]pyrene, indeno[1,2,3,-cd]pyrene, dibenzo[a,h]anthracene, and benzo[g,h,i]perylene). Aliphatic hydrocarbons with C > 12 were determined by gas chromatography equipped with a flame ionization detector (GC-FID), according to the method proposed by the Italian Institute for Environmental Protection and Research (Balzamo et al., 2012). The concentrations of arsenic, cadmium, chromium, nickel, lead, and zinc in the sediments were determined after microwave-assisted acid digestion with a mixture of HNO_3_, HF, H_2_O_2_ (EPA 3052) and analyzed by inductively coupled plasma-mass spectrometry (ICP-MS). Iron concentrations were analyzed by inductively coupled plasma-optical emission spectrometry (ICP-OES), after the same acid digestion. Mercury concentrations were analyzed by Atomic Absorption Spectrometry AMA-254 (EPA 7473; Armiento et al., 2020). For both heavy metals and PAHs, the effect range median quotients (m-ERM-q; Kowalska et al., 2018) were calculated based on the available ERM values for these contaminants, to assess their potential toxicity on benthic biota (Long et al., 1995).

### Grain Size and Quantity and Biochemical Composition of Organic Matter

Sediment grain size was determined by the sieving technique (Danovaro, 2010). Samples were treated with a 10% H_2_O_2_ solution to remove organic matter, and the total and non-biogenic grain size distribution was determined. The concentrations of chlorophyll-a and phaeopigments in the sediments were determined fluorometrically, according to standard protocols and their sum referred to as phytopigment concentrations (Danovaro, 2010). Protein, carbohydrate, and lipid concentrations were determined spectrophotometrically, following the protocols detailed in Dell’Anno et al. (2002), and their sedimentary contents expressed as bovine serum albumin, glucose and tripalmitin equivalents, respectively. Carbohydrate, protein, and lipid sedimentary contents were converted into carbon equivalents using the conversion factors of 0.40, 0.49, and 0.75 μgC μg^−1^, respectively, and their sum was defined as biopolymeric carbon (BPC; Dell’Anno et al., 2002).

### Prokaryotic Abundance

Prokaryotic abundance was determined after cell detachment from sediment using tetrasodium pyrophosphate (final concentration, 5 mM) and ultrasound treatment (three 1-min treatments) by using a Branson Sonifier 2,200 (60 W) and properly diluted with sterile and 0.2-μm-prefiltered formaldehyde solution (2% final concentration; Danovaro, 2010). Sub-aliquots were stained with SYBR Green I and filtered on Anodisc filters (pore size, 0.2 μm). Filters were analyzed by epifluorescence microscopy (1,000 × magnification). For each slide at least 10 fields were observed and a total of at least 400 cells were counted (Danovaro, 2010).

### Diversity and Taxonomic Composition of the Benthic Prokaryotic Assemblages

To assess the diversity and taxonomic composition of the prokaryotic assemblages in the sediments of the four sites, the microbial genomic DNA was extracted from the sediments using the DNeasy PowerSoil Kit (Qiagen) with some modifications for the removal of extracellular DNA (Danovaro, 2010). The extracted DNA was sequenced by Illumina MiSeq (V3 technology; 2 × 300 bp), with primers targeting the V4-V5 hypervariable regions (Parada et al., 2016) at LGC Genomics GmbH (Berlin, Germany), generating a total of 1,529,783 paired-end reads (152,978,30 ± 42,419,28, on average). Raw paired-end reads were joined using the BBMerge tool from the BBMap suite (Bushnell, 2014) in a two-step process: reads that did not merge in a first step were quality-trimmed to remove low-quality bases (*Q* < 10) prior to re-joining to increase the number of merged sequences. Subsequently, joined sequences were analyzed using the QIIME2 pipeline^1^ following a previously published pipeline (Bolyen et al., 2019). Amplicon sequence variants (ASVs) were identified using the DADA2 strategy (Callahan et al., 2016). The SILVA database v138 (Quast et al., 2012) was used as a reference database for taxonomic affiliation of sequences; briefly, reference 16S sequences contained in the database were trimmed within QIIME2 to the region amplified by sequencing primers and representative ASVs were analyzed using the classify-consensus-vsearch approach (consensus over 51% of at most five best hits) for taxonomic affiliation (Rognes et al., 2016). Classes and families were defined “rare” when their average contribution across all samples was lower than 1%. The final ASV abundance table was then rarefied at 50,000 sequences per sample through the alpha-rarefaction subcommand in QIIME2 (Bolyen et al., 2019). We checked that all rarefaction curves reached a plateau using this number of sequences, which allowed us to obtain a robust estimate of the actual bacterial diversity in the investigated samples (Supplementary Figure S1). Moreover, we utilized the core-metrics-phylogenetic subcommand to contextually calculate the ASV richness, Pielou’s index, Shannon index, and Faith’s phylogenetic diversity index for all samples, as well as to produce a rarefied ASV table, which was used for downstream statistical analyses.

### Isolation and Screening of Bacterial Strains From the Contaminated Sediments

Sub-aliquots of the freshly-collected contaminated sediments were used to isolate bacterial strains in petri dishes containing Marine Agar (MA; Bacto-Agar, Difco) and different concentrations of PAHs (from 0 up to 20,000 ppm for single congener represented by naphthalene, phenanthrene, and pyrene; i.e., up to concentrations ~100 times higher than the highest concentrations of each PAH congeners found in the investigated sediments). The bacterial colonies grown at the highest PAHs concentrations were subsequently screened for their contextual ability to tolerate high concentrations of HMs, by means of minimum Inhibition concentration (MIC) tests (Andrews, 2001). Briefly, the bacterial isolates selected in the PAHs-enriched media were inoculated in liquid MA at a final concentration of 10^8^ cells/ml (determined by TECAN M1000 spectrophotometric analysis), and then transferred in replicate plates. Once solid, each MA plate was then punctured with a pipette tip and the resulting small hole (approx. 2 mm diameter) was spotted with 10 μl of each metal (either As or Pb, Zn, Cd, and Cu) at five different concentrations (2, 20, 200, 2,000, and 20,000 ppm), up to concentrations ~100 times higher than the maximum concentration of HMs found in the investigated sediments. After 48 h of incubation at 28°C, the diameter of each halo of inhibition was measured, if present. For each plate, spotting with antibiotic mix (penicillin and streptomycin, 50 mg/ml final concentration) was used as positive control (i.e., providing a standard 100% inhibition). The MIC was estimated as the lowest concentration of a metal which prevented visible bacterial growth (Andrews, 2001), and the level of inhibition was expressed as percent inhibition relative to the antibiotic-inhibited positive control. For each bacterial strain, all treatments were performed in triplicates.

### Identification of the Bacterial Isolates Based on 16S rRNA Gene Sequencing

The DNA of the bacterial isolates was extracted using the Qiagen Blood and Tissue Kit, according to the manufacturer’s instructions, and the 16S rRNA gene was PCR-amplified using the MasterTaq® kit (Eppendorf AG, Germany) and a Biometra PCR thermal cycler (Germany). Each 50-μl reaction contained 10 ng of genomic DNA, 1x PCR buffer, 200 μM of each dNTP and 0.5 μM of each primer (27F-5'AGAGTTTGATCMTGGCTCAG and 907R-5'CCGTCAATTCMTTTRAGTTT). PCR included 25 cycles of 95°C for 30 s, 55°C for 45 s and 72°C for 90 s, preceeded by 5 min of denaturation at 94°C and followed by a final extension of 10 min at 72°C. The amplicons were checked on agarose-Tris-borate-EDTA (TBE) gels (1%), containing GelRED™ (Biotium, United States) for DNA staining and visualization, purified and Sanger-sequenced (Macrogen Inc.). The consensus sequences of the isolates were compared with those available in the SILVA database using the ACT aligner provided on the web server (Pruesse et al., 2012) and the database automatically queried to retrieve closest neighbors with minimum seq. identity of 80% for each query sequence. The 16S rRNA gene sequences retrieved from the databases were aligned using the MUSCLE algorithm (Edgar, 2004) included in the MEGA software, version 4.1 (Hall, 2013). Evolutionary analysis was inferred by the Maximum Likelihood method using default settings (Yang, 2007). The Sanger sequencing-derived 16S sequences of cultured isolates were co-aligned using MAFFT with ASVs representative sequences. ASVs with similarity >97% to one of the isolate sequences within the amplified region were kept and classified using the SILVA database (v138) on the SILVA website.

### Statistical Analyses

To test for differences of the investigated variables among the different benthic sampling sites permutational analyses of variance (PERMANOVA) were carried out. For all analyses, when significant effects were observed, pair-wise tests were also carried out (Anderson et al., 2008). To test the potential effects of environmental variables, chemical contaminants, and trophic resource availability on prokaryotic assemblage composition, non-parametric multivariate multiple regression analyses based on Euclidean distances were carried out, using the DISTLM routine and the forward selection procedure with *R*^2^ as the selection criterion and 4,999 permutations (McArdle and Anderson, 2001). To run this test, the prokaryotic assemblage composition was used as dependent variable (using the rarefied ASV table as input), whereas potential explanatory variables included biopolymeric C, phytopigments, PAH, aliphatic hydrocarbon, and HM concentrations, grain size, pH, and Eh. All statistical tests were performed using the routines included in the PRIMER-6 and PERMANOVA software (Clarke and Gorley, 2006).

## Results and Discussion

The four benthic sites of Bagnoli-Coroglio were characterized by a wide variability of the different environmental and chemical variables investigated (Table 1). In particular, the sediments of site S1 displayed the highest concentrations of aliphatic hydrocarbons (126.5 μg g^−1^) and of Cr (31.1 μg g^−1^). Site S2 was characterized by the highest concentrations of As (120.7 μg g^−1^), but the lowest concentrations of phytopigments and BPC (1.1 μg g^−1^ and 0.6 mg g^−1^, respectively), of aliphatic hydrocarbons (31.0 μg g^−1^) and of Cd, Cu, Hg, and Ni (0.24, 7.4, 0.028, and 8.3 μg g^−1^, respectively). The highest concentrations of phytopigments and BPC (4.5 μg g^−1^ and 6.3 mg g^−1^, respectively), as well as of PAHs (305.8 μg g^−1^) and of Cu, Hg, Ni, Pb, Zn, and Fe (14.2, 0.12, 11.4, 105.7, 323.2, and 58,356 μg g^−1^, respectively), were observed in the sediments of site S3. Site S4 displayed the highest sediment concentrations of Cd (0.63 μg g^−1^), but the lowest for PAHs (3.8 μg g^−1^) and for As, Pb, Zn, and Fe (17.2, 67.3, 105.6, and 26,933 μg g^−1^, respectively). The sediment grain size distribution differed largely among sites, with S1 and S2 displaying an overall finer grain size (>88% below 0.5 mm) compared with the coarser grain size of the sediments of the S3 and S4 sites (>66% above 0.5 mm). The investigated sites differed also in terms of sediment pH (from 6.79 in S2 to 8.03 in S4) and Eh (from 167 in S4 to 264 in S2).

**Table 1:** Environmental, chemical and microbiological variables at the four benthic stations of the Bagnoli-Coroglio area.

		S1 (Dazio)	S2 (Agnano)	S3 (Bianchettaro)	S4 (Coroglio)
Latitude		40.816281	40.815249	40.809144	40.796925
Longitude		14.159003	14.162204	14.166972	14.176825
Phytopigments (μg g^−1^)		2.1 ± 0.1	1.1 ± 0.1	4.5 ± 1.7	4.4 ± 0.6
Biopolymeric C (mg g^−1^)		0.7 ± 0.2	0.6 ± 0.1	6.3 ± 3.2	1.1 ± 0.2
Prokaryotic abundance (10^7^ cells g^−1^)		3.7 ± 0.3	3.4 ± 1	2.6 ± 0.3	1.9 ± 0.1
pH		7.41	6.79	8.02	8.03
Eh		178	264	197	167
Polycyclic aromatic hydrocarbons (PAHs; μg g^−1^)		8.3	5.5	305.8	3.8
Aliphatic hydrocarbons (C > 12; μg g^−1^)		126.5	31.0	39.5	81.8
As (μg g^−1^)		92.1	120.7	22.4	17.2
Cd (μg g^−1^)		0.36	0.24	0.36	0.63
Cr (μg g^−1^)		31.1	27.1	15.3	28
Cu (μg g^−1^)		10.9	7.4	14.3	13.5
Hg (μg g^−1^)		0.052	0.028	0.12	0.034
Ni (μg g^−1^)		10.4	8.3	11.4	8.6
Pb (μg g^−1^)		85.9	76.8	105.7	67.3
Zn (μg g^−1^)		215.1	160.9	323.2	105.6
Fe (μg g^−1^)		55,407	44,692	58,356	26,933
m-ERM-q for PAHs		0.44	0.28	15.37	0.18
m-ERM-q for Heavy Metals		0.33	0.35	0.26	0.15
Sediment texture (grain size %)	>2 mm	0.1 ± 0.08	0.04 ± 0.02	36.33 ± 6	3.01 ± 1.06
	1–2 mm	0.8 ± 0.1	0.51 ± 0.12	21.26 ± 3.06	12.9 ± 4.93
	0.5–1 mm	10.6 ± 1.0	8.5 ± 2.5	24.7 ± 2.2	49.8 ± 13.6
	0.25–0.5 mm	37.4 ± 2.0	52.8 ± 4.2	13.1 ± 1.3	27.8 ± 14.4
	0.125–0.25 mm	47.3 ± 2.9	36.7 ± 6.4	4.0 ± 0.4	6.2 ± 5.0
	0.063–0.125 mm	3.7 ± 0.4	1.3 ± 0.3	0.3 ± 0.1	0.2 ± 0.1
	<0.063 mm	0.05 ± 0.01	0.07 ± 0.02	0.3 ± 0.04	0.1 ± 0.04

The m-ERM-q values calculated for HMs and PAHs (Long et al., 1995; Long and Macdonald, 1998; Kowalska et al., 2018), highlighted a medium level of potential ecotoxicological risk at sites S1, S2, and S4, and a high risk, due to the particularly high concentrations of PAHs, at site S3 (Table 1).

Overall, these results confirm a high level of contamination in this area as previously reported (Romano et al., 2004; Arienzo et al., 2017; Trifuoggi et al., 2017, Armiento et al., 2020; Morroni et al., 2020; Tangherlini et al., 2020), and highlight a high variability in the mixtures of contaminants and environmental characteristics, even at a small spatial scale.

The prokaryotic abundances at the four shallow-water benthic sites investigated in this study were lower compared to those previously reported at greater water depths in the Bagnoli-Coroglio (Table 1; Tangherlini et al., 2020), but they were comparable to the values observed in different unpolluted coastal benthic sites of the Mediterranean Sea (Dell’Anno et al., 2003; Quero et al., 2015). These results reinforce previous findings that the chemical contaminants present in the sediments of the Bagnoli-Coroglio area do not significantly affect the autochthonous microbial assemblages in terms of prokaryotic standing stocks (Tangherlini et al., 2020).

**Figure 2:**
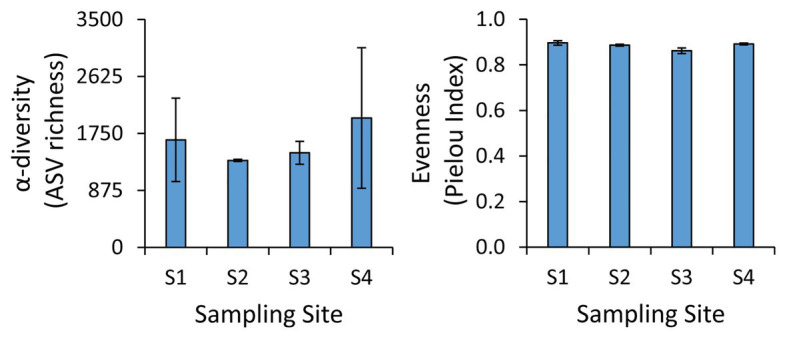
Values of α-diversity and evenness at the benthic sites investigated. Average values and SD are reported.

The taxonomic analysis of the prokaryotic assemblages based on next-generation sequencing of the 16S rRNA genes revealed the lack of significant differences in the α-diversity and evenness values (Pielou index) among the four sampling sites (Figure 2). In particular, we found an average α-diversity of 1,606 ± 599 ASVs, with values ranging from 1,130 ASVs at site S1 to 3,217 ASVs at site S4, while the Pielou Index varied from 0.86 at site S3 to 0.91 at site S1 (Figure 2). Such values of ASV richness and evenness are similar to those found in the benthic sites at greater water depth in the same area (Tangherlini et al., 2020), and they are in the range of those found also in non-polluted benthic coastal systems (Quero et al., 2015; Wambua et al., 2019; Basili et al., 2020). These results suggest that the different environmental settings and the different levels and types of contaminants found in the four sites did not exert a major influence on the richness of prokaryotic taxa or on their evenness.

**Figure 3:**
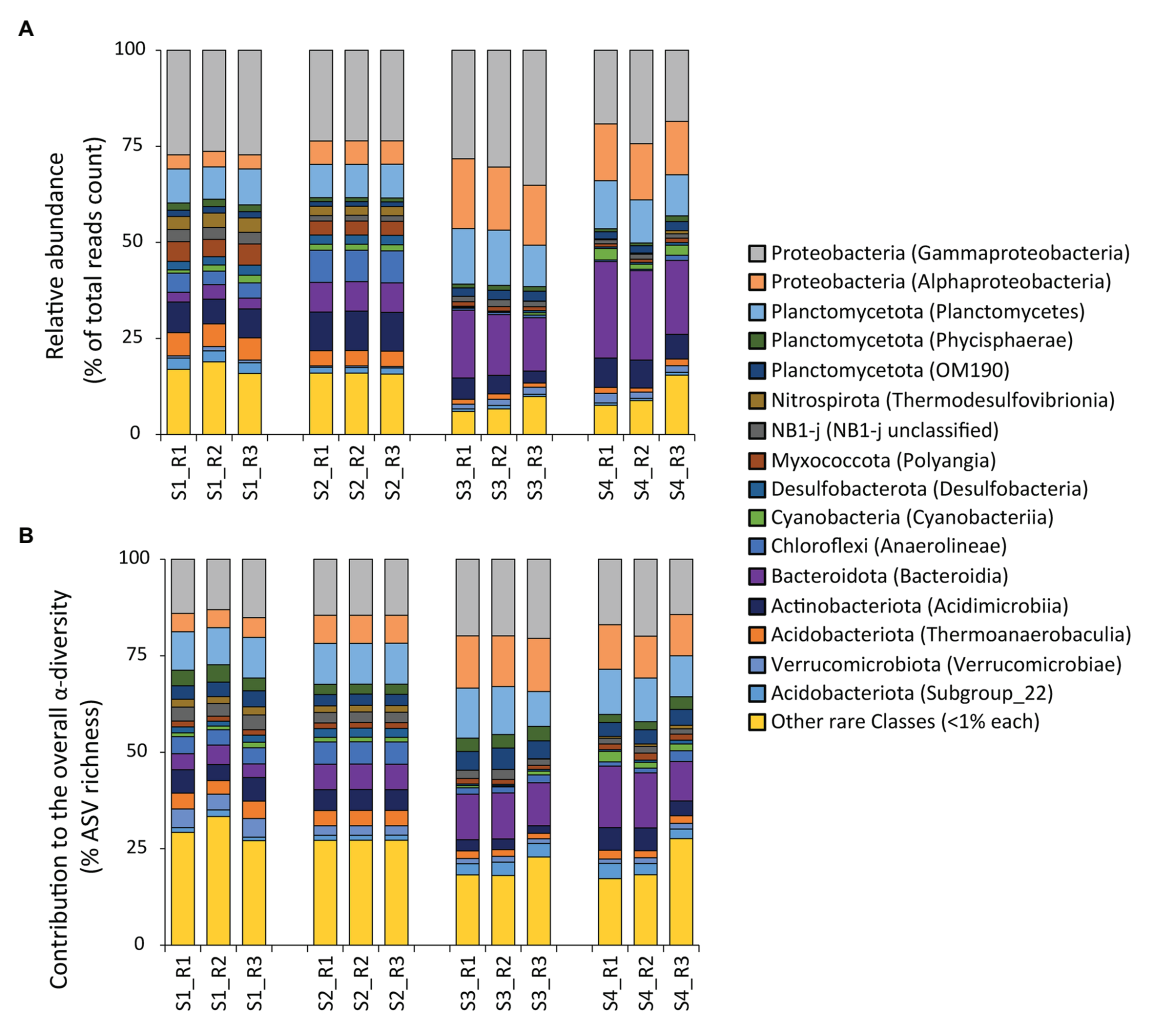
**(A,B)** Composition of the prokaryotic assemblages at the benthic sites investigated. Reported are: **(A)** the relative abundance of each identified prokaryotic class (as % contribution to the total reads count), and **(B)** the contribution of each class to the overall α-diversity in terms of number of amplicon sequence variant (ASV) compared with the total ASV richness (expressed as percentage).

The taxonomic annotation of the ASVs allowed us to identify 61 phyla and 146 classes. The phylum Proteobacteria dominated at all sites, contributing from 30 to 48% to the total reads counts (with min. values at site S2 and max. values at site S3; Figure 3A). Other abundant phyla were Planctomycetota (from min. 12% to max. 18% of the total reads counts, respectively at site S2 and at site S3), Bacteroidota (from min. 4% at S1 to max. 23% at S4), Acidobacteriota (from min. 2% at S3 to max. 11% at S1), and Actinobacteriota (from min. 5% at S3 to max. 10% at S2). In all samples, the top five most abundant classes were Gammaproteobacteria (from 21% at S4 to 31% at S3), Bacteroidia (from 3% at S1 to 23% at S4), Alphaproteobacteria (from 4% at S1 to 17% at S3), Planctomycetes (from 9% at S1 and S2 to 13% at S3), and Acidimicrobiia (from 4% at S3 to 10% at S2; Figure 3A). Such bacterial assemblage structure is largely in accordance with those found in other coastal sediments worldwide affected by hydrocarbon and heavy metal contamination (Sun et al., 2013; Quero et al., 2015 and references therein). A previous study conducted to investigate the influence of chemical contaminants on microbial assemblages in the sediments of this same bay, found that the most represented prokaryotic classes were Gammaproteobacteria, Deltaproteobacteria, Bacteroidia, Nitrososphaeria (mainly, Thaumarchaeota), and Alphaproteobacteria, with a high representation by unclassified bacterial taxa (Tangherlini et al., 2020). In our study, we confirmed the relevance of Gammaproteobacteria, Alphaproteobacteria, Bacteroidia, and Deltaproteobacteria (recently re-classified with the use of v138 instead of v132 SILVA database mainly as NB1-j, Desulfobacterota, and Myxococcota; Figure 3A; Waite et al., 2020). Conversely, we found a much lower representation by Thaumarchaeota, likely due to fact that the sediment samples analyzed in Tangherlini et al. (2020) were collected at deeper water depth, where Thaumarchaeota are typically more abundant (Massana et al., 1997; Karner et al., 2001; Tolar et al., 2013).

As the rare classes (i.e., those with average <1% contribution across all samples) accounted overall for a consistent fraction of the overall reads counts in our study (average, 13 ± 5%, from min. 8% at S3 to max. 17% at S1), we analyzed this portion of the bacterial diversity at higher-level taxonomic resolution (Supplementary Figure S2). This analysis revealed that a total of seven classes were actually not rare locally (i.e., >1% contribution in at least one site). In detail, the comparison across sites highlighted a higher contribution of Latescibacterota, AKAU4049 and BD2-11 at site S1, while of Ignavibacteria and Calditrichia at S2, of Bdellovibrionia at S3, and of Desulfobulbia at S4 (Supplementary Figure S2). Members of all these classes are abundant in environments rich in HMs and/or hydrocarbons, suggesting their quantitative and possibly functional relevance in marine sediments that display high concentrations of such contaminants (Lanoil et al., 2001; Iino et al., 2010; Youssef et al., 2015; Cerqueira et al., 2017; Bergo et al., 2020; Jin et al., 2020; Costa et al., 2021). In particular, the differences in the relative contribution of these taxa across the sites of the Bagnoli Coroglio Bay suggest that the specific contaminants present at each benthic site might contribute to determine a local increase of bacterial taxa, which are elsewhere rare.

**Figure 4:**
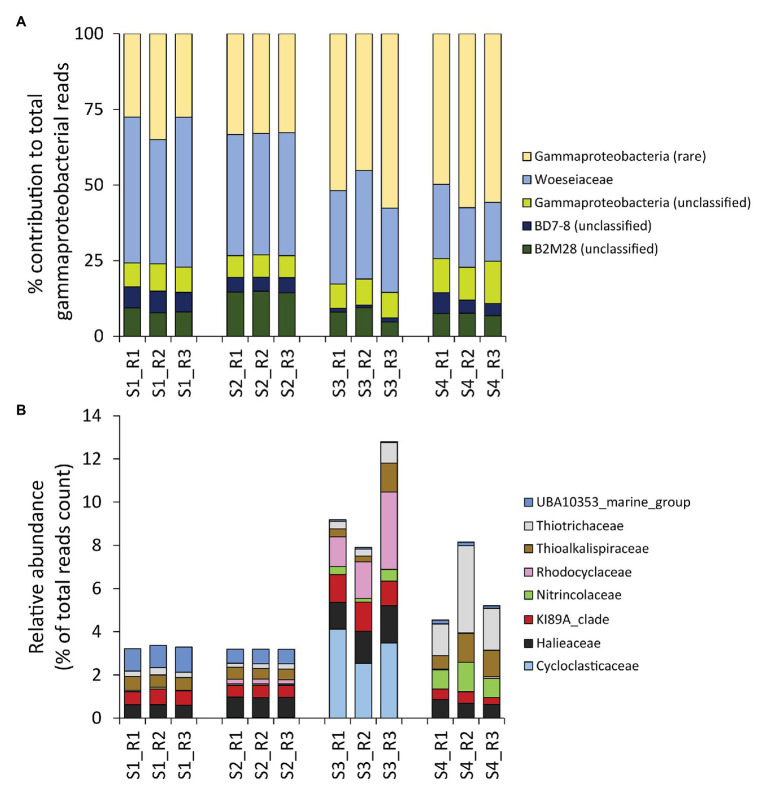
**(A,B)** Composition of benthic bacterial assemblages affiliated to the class Gammaproteobacteria. Reported is **(A)** the contribution of each identified gammaproteobacterial Family within the Gammaproteobacteria Class (expressed as percentage), and **(B)**, a highlight on the eight gammaproteobacterial families that resulted rare within the dataset (i.e., on average contributing for less than 1% to the overall reads count across samples), but displayed peaks with relative contributions >1% in some samples.

Despite the contribution of Gammaproteobacteria to the total reads was twice as high as that of the rare classes combined (on average, 26 ± 5 vs. 13 ± 5%, respectively), gammaproteobacterial taxa were less diverse, exhibiting a number of ASVs much lower than that of the rare classes combined (on average, 16 ± 3 vs. 24 ± 5%, respectively; Figure 3B). Moreover, even within the Gammaproteobacteria, the rare gammaproteobacterial taxa (i.e., contributing each for less than 1% of the total reads counts) represented a large portion (42 ± 12%) of the overall reads affiliated to Gammaproteobacteria (Figure 4A).

Overall, these results highlight the high contribution of rare taxa to the total prokaryotic diversity in the sediments of the sites investigated in this study. This observation is consistent with the relatively high values of evenness found in all samples investigated (average Pielou index of 0.88 ± 0.02), similar to values obtained at a wider spatial scale in the same area (0.93 ± 0.03; Tangherlini et al., 2020).

To gain a deeper insight on the rare portion of the gammaproteobacterial diversity, we conducted a higher-level taxonomic resolution analysis, which indicated that a total of eight gammaproteobacterial families were actually not rare locally (i.e., >1% contribution in at least one site), including Cycloclasticaceae, Halieaceae, KI89A clade, Nitrincolaceae, Rhodocyclaceae, Thioalkalispiraceae, Thiotrichaceae, and UBA10353 marine group (Figure 4B). All of these families include members recently reported to be particularly abundant in naturally-rich and/or contaminated environments that display high concentrations of HMs and/or hydrocarbons (Gołębiewski et al., 2014; Singleton et al., 2015; Yang et al., 2016, 2020; Suzuki et al., 2019; Bergo et al., 2020; Noirungsee et al., 2020; Sun et al., 2020). Notably, Cycloclasticaceae, Halieaceae, and Rhodocyclaceae, well known for their ability to degrade hydrocarbons, showed a peak in the site most contaminated by PAHs (S3; Figure 4B; Table 1).

Overall, these results suggest that the rare bacterial taxa in the sediments of the Bagnoli-Coroglio bay are particularly important not only in terms of contribution to the overall bacterial diversity, but also for their potential interplay with the sediment contaminants, with peaks of different taxa at the different sites.

The DISTLM analysis showed that the dominant taxa were influenced by different factors compared with the rare taxa (Supplementary Table S1). Indeed, the distribution of the dominant taxon (Gammaproteobacteria) was mainly influenced by PAHs, As, and Cd concentrations, and that of the other abundant taxa (including Alphaproteobacteria, Bacteroidetes, Acidobacteria, Actinobacteria, NB1-j, Desulfobacterota, and Myxococcota) by sediment grain size and by Cu and Cr concentrations. Conversely, the distribution of the rare taxa was mainly influenced by As and aliphatic hydrocarbon concentrations and by sediment redox potential. These results suggest a differential response of each bacterial taxon to environmental features and chemical contamination and that specific bacterial taxa might be inhibited or stimulated by different contaminants. These findings have important implications for the selection of autochthonous bacterial strains to be used in the bioremediation of sediments that display mixtures of different contaminants. Indeed, our results suggest that the bacterial taxa potentially able to degrade PAHs in the sediments may be inhibited by the contextually high concentrations of HMs, in turn, excluding the possibility of using these strains successfully in the degradation of PAHs for sediment bioremediation (Thompson et al., 2005; Tyagi et al., 2011; Dueholm et al., 2015). We thus focused our attention to search for optimal candidates for sediment bioremediation purposes by culturing and identifying bacterial strains able to grow contextually in the presence of high concentrations of PAHs and HMs.

The plating of contaminated sediment samples onto PAHs-enriched media led to the isolation of 80 different bacterial strains able to grow in the presence of 60,000 ppm of PAHs (as the sum of naphthalene, phenanthrene, and pyrene). All of these 80 isolates were easy to isolate/grow and rapidly reached an OD_600_ of 2.5–3 (corresponding to approximately 3–5 × 10^9^ cells ml^−1^) after 1 day, showing no inhibition by PAHs. The subsequent screening of these colonies based on their ability to maintain similarly high growth rates at high HMs concentrations led to the selection of nine colonies. Specifically, we obtained five isolates (named ABB.1, ABB.2, ABB.3, ABB.6, and ABB.7) from the sediments collected at site S4, two isolates (ABB.10 and ABB.15) from site S1, one isolate from S2 (ABB.9) and one from site S3 (ABB.12).

**Figure 5:**
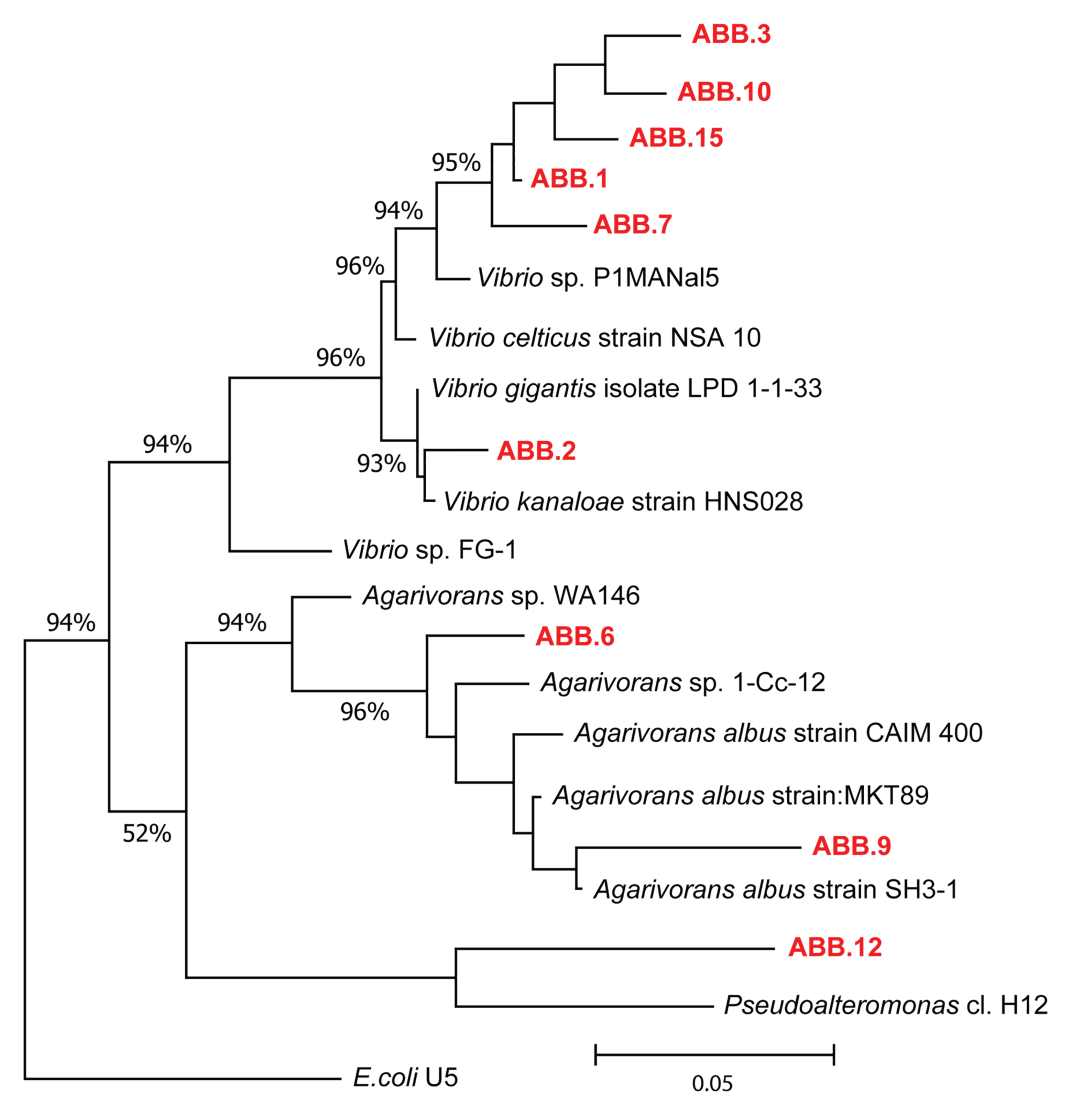
Phylogenetic tree of the nine bacterial strains isolated from the sediments of the Bagnoli-Coroglio area. The tree shows the affiliations of the nine bacterial strains (highlighted in red, ABB.1, 2, 3, 6, 7, 9, 10, 12, and 15) based on 16S rRNA gene sequencing.

**Figure 6:**
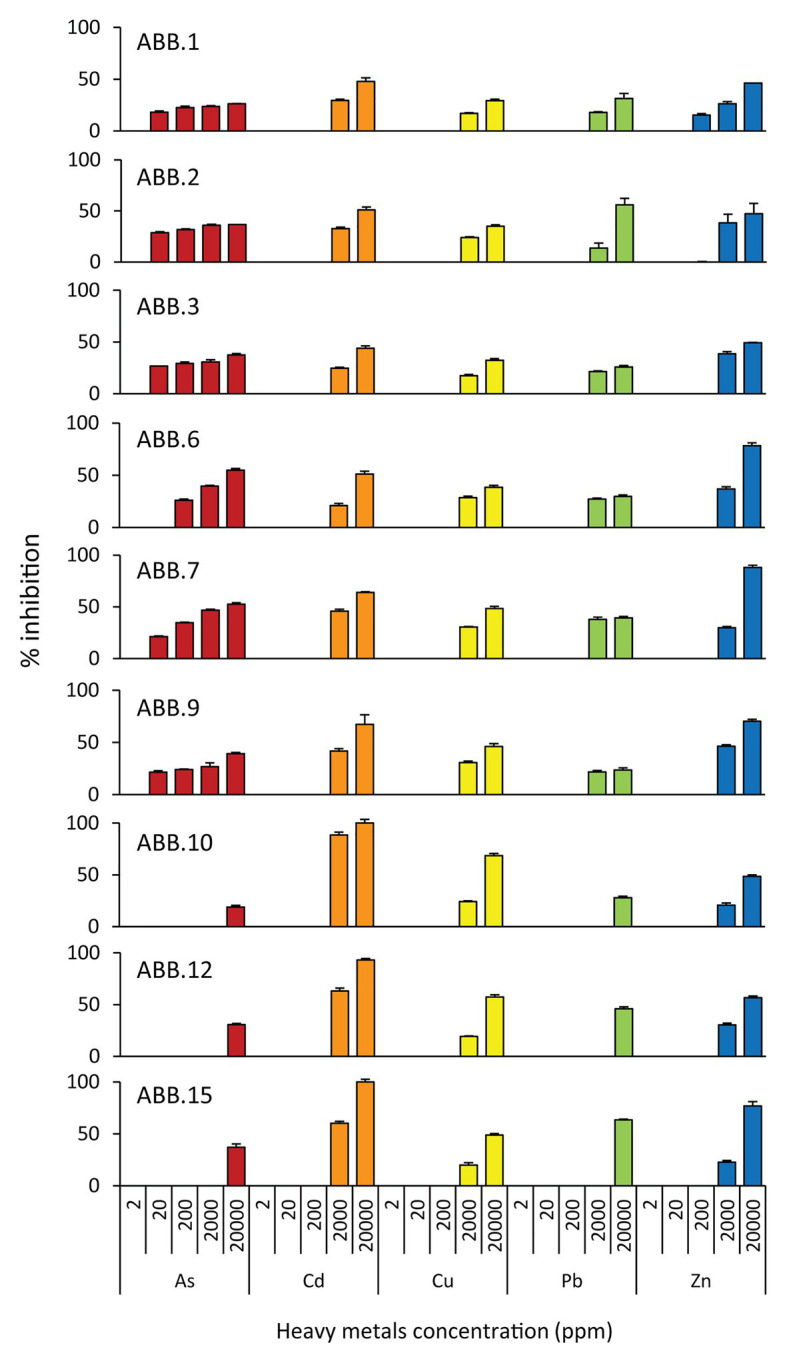
Resistance of the isolated bacterial strains to heavy metals (HMs). Reported is the percentage of inhibition (from 0 to 100%) of the nine bacterial strains (ABB.1, 2, 3, 6, 7, 9, 10, 12, and 15) to the different HMs tested at different concentrations (from 0 to 20,000 ppm).

**Figure 7:**
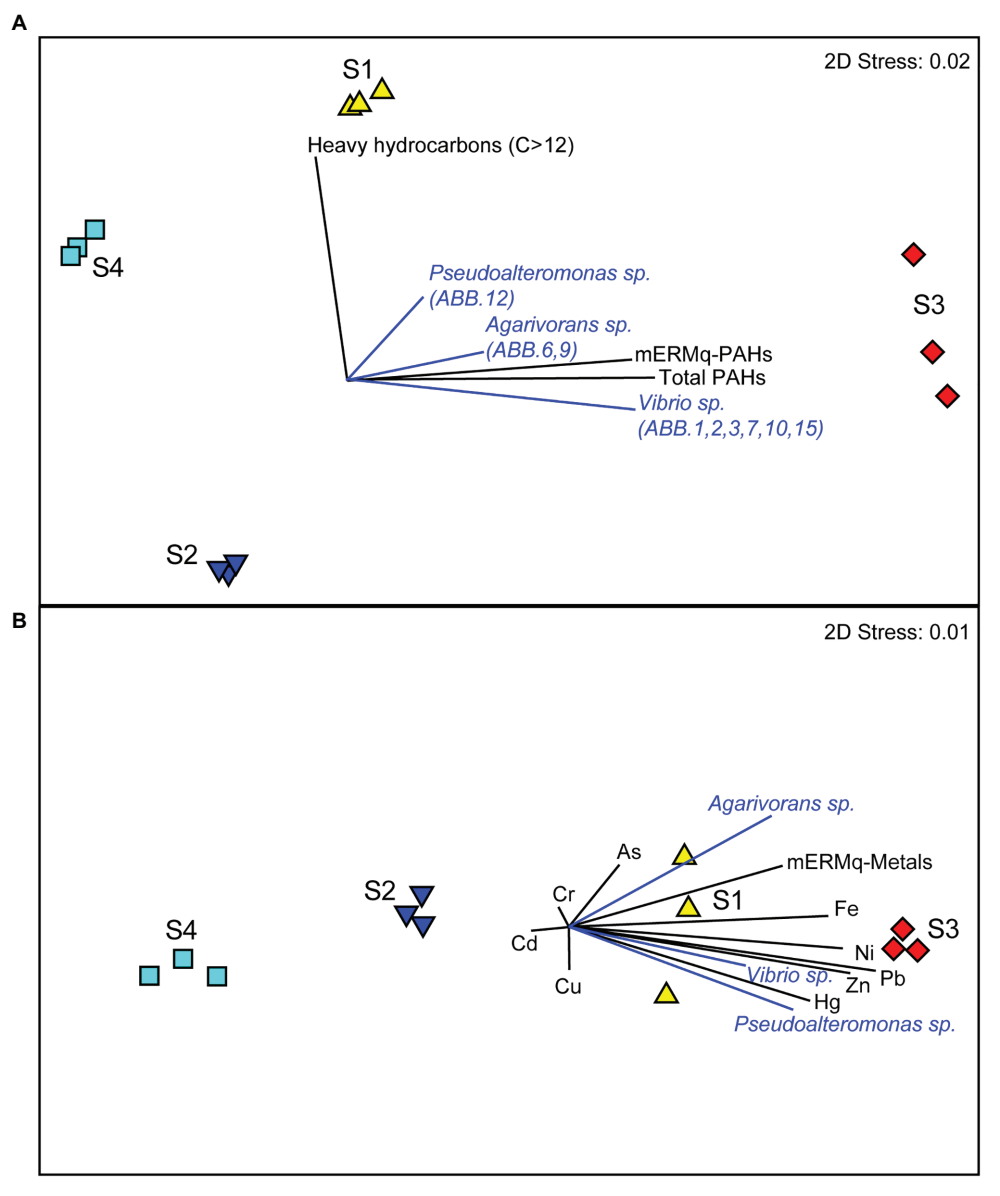
**(A,B)** Relationship between the organic and inorganic contaminations and the distribution of *Vibrio* sp., *Agarivorans* sp. and *Pseudoalteromonas* sp. in the investigated benthic sites of the Bagnoli-Coroglio area. **(A)** The MDS plot shows the distribution of the sediment samples based on aliphatic hydrocarbon and polycyclic aromatic hydrocarbon (PAH) contamination levels, with black vectors indicating higher values of hydrocarbon contamination and of the resulting mEMq values for PAHs at sites S1 and S3, and blue vectors indicating the higher contribution of the sequences affiliated to *Vibrio* sp., *Agarivorans* sp., and *Pseudoalteromonas* sp. isolates to the overall prokaryotic assemblage. **(B)** The MDS plot shows the distribution of the sediment samples based on HMs contamination levels, with black vectors indicating higher values of the different HMs and of the resulting mEMq values for HMs at sites S1 and S3, and blue vectors indicating the higher contribution of the sequences affiliated to *Vibrio* sp., *Agarivorans* sp., and *Pseudoalteromonas* sp., isolates to the overall prokaryotic assemblage.

The nine bacterial isolates affiliated to Gammaproteobacteria. In particular, six isolates affiliated to *Vibrio* (ABB.1, ABB.2, ABB.3, ABB.7, ABB.10, and ABB.15), two to *Agarivorans* (ABB.6 and ABB.9) and one to *Pseudoalteromonas* (ABB.12; Figure 5). None of the nine bacterial isolates shared identical 16S rRNA gene sequence with their respective best match in GeneBank. While the *Vibrio* and *Agarivorans* strains obtained in this study displayed 97–99% identity with their best match, ABB.12 shared only 82% identity with its best reference sequence (uncultured *Pseudoalteromonas* clone GeneBank id. FR848714), suggesting that this particular isolate could represent a novel bacterial lineage. All of the nine bacterial isolates selected in our study displayed a wide resistance to high HMs concentrations and were completely unaffected by concentrations of Cd, Cu, Pb, and Zn up to 200 μg g^−1^ (Figure 6), with low levels of inhibition also by As concentrations as high as 2,000 μg g^−1^ (growth inhibition always <50%; Figure 6). These results indicate that these nine isolates can grow at HM concentrations well above those found in their respective original sediments (Table 1). We could highlight the two strains ABB.1 and ABB.3, which showed low percentages of inhibition for all HMs at the highest concentrations tested (<50% growth inhibition at 20,000 μg g^−1^ of all HMs; Figure 6). Moreover, we found ABB.10, ABB.12 and ABB.15 to be the most tolerant to HMs, being completely unaffected by Cd, Cu, and Zn concentrations up to 200 μg g^−1^ and by As and Pb concentrations as high as 2,000 μg g^−1^ (Figure 6). Notably, ABB.10, ABB.12, and ABB.15 were isolated from sites S1 and S3, which were the most contaminated with aliphatic hydrocarbons, PAHs, as well as several HMs (Hg, Ni, Pb, Zn, and Fe), suggesting that these strains could be highly adapted to cope with such harsh conditions present in the sediments.

The comparison of the results obtained by the metabarcoding of the 16S rRNA genes of the benthic prokaryotic assemblages with those obtained by the sequencing of the 16S rRNA genes of the isolated bacterial strains allowed investigating the representation of the isolated strains in the natural benthic microbial assemblages. The 16S rDNA sequences of all nine strains were only scarcely represented in the overall metabarcoding sequence output (i.e., only three ASVs affiliating to *Vibrio*, one to *Pseudoalteromonas* and one to *Agarivorans*, overall contributing less than 0.1% of the total reads in all samples), indicating that these strains were very rare in the natural assemblages. Moreover, we found that the relative contribution of sequences affiliated to *Vibrio* sp., *Agarivorans* sp., and *Pseudoalteromonas* sp. isolates to the overall prokaryotic assemblages was higher at sites S1 and S3 and positively correlated with aliphatic hydrocarbon and PAH concentrations (Figure 7A), as well as with several HMs (Hg, Ni, Pb, Zn, and Fe; Figure 7B). These results suggest that these strains could be favored by hydrocarbon contamination and may actively contribute to the degradation of aliphatic hydrocarbons and PAHs in the sediments of Bagnoli-Coroglio area, despite the high HMs concentrations.

To our knowledge, our study suggests for the first time a potential for members of the genus *Agarivorans* to contribute to bioremediation processes in marine sediments (López-Pérez and Rodriguez-Valera, 2014). Conversely, members of the genera *Vibrio* and *Pseudoalteromonas* have been reported several times in the literature for their ability (based on experimental evidence) or potential (based on genomic information) to degrade hydrocarbons (Zhuang et al., 2003; Hedlund and Staley, 2006; Al-Awadhi et al., 2012; Hu et al., 2015; Radwan and Al-Mailem, 2015; Lee et al., 2018a,b). Moreover, *Vibrio* and *Pseudoalteromonas* also include members involved in HMs detoxification/remediation (Yu et al., 2013; Sowmya et al., 2014; Jafari et al., 2015; Saranya et al., 2017), thus further supporting the potential role of the strains identified in this study in the bioremediation processes in the sediments of the Bagnoli-Coroglio bay. The nine bacterial strains identified in our study showed several of the characteristics required to be considered good candidates for effective bioremediation, as discussed in the Introduction section (Mohajeri et al., 2017; Dell’Anno et al., 2018; Mahjoubi et al., 2018; Singh et al., 2020). Indeed, they were resistant to mixed contaminants and they were easy to isolate/grow. At the same time, they could be stimulated to produce a large biomass if supplemented with adequate amounts of nutrients, thus showing good potential both for biostimulation and bioaugmentation approaches, which could overcome eventual limitations due to the low abundance of these strains in the original sediments.

Overall, the results presented in this study indicate that the use of an integrated approach, based on the analysis of the bacterial taxa through high-throughput sequencing and culturing, can be a promising strategy to identify autochthonous bacterial strains potentially useful for sediment bioremediation. Indeed, this approach may be of great interest for bioaugmentation applications aimed at the reclamation of marine sediments that are highly contaminated with PAHs and HMs.

## Data Availability Statement

The datasets presented in this study can be found in online repositories. The names of the repository/repositories and accession number(s) can be found at: https://figshare.com/s/78a47c560d6fa2b1f19b.

## Author Contributions

AD, ER, and CS conceived the study. FD, ER, CS, and LM conducted the field work. FD, ER, MT, and MM conducted the laboratory analyses. FD, ER, MT, CS, CB, SB, and AD contributed to data elaboration. FD and ER wrote the draft of the manuscript. All authors contributed to the article and approved the submitted version.

### Conflict of Interest

The authors declare that the research was conducted in the absence of any commercial or financial relationships that could be construed as a potential conflict of interest.

## Footnotes

^1^https://qiime2.org
