# Supplementary material for: Highly Contaminated Marine Sediments Can Host Rare Bacterial Taxa Potentially Useful for Bioremediation
Source: Front Microbiol. 2021 Mar 1;12:584850. doi: 10.3389/fmicb.2021.584850 (PMC7956957; doi:10.3389/fmicb.2021.584850)
Supplement: Supplementary file 1 [file Data_Sheet_1.docx]

**Supplementary material**

**Highly contaminated marine sediments can host rare bacterial taxa potentially useful for bioremediation**

F. Dell’Anno^1^, E. Rastelli^1^, M. Tangherlini^1^, C. Corinaldesi^2^, C. Sansone^1^, C. Brunet^1^, S. Balzano^1^, A. Ianora^1^, L. Musco^1^, M.R. Montereali^3^, A. Dell’Anno^4^

*^1^Stazione Zoologica Anton Dohrn, Villa Comunale, Naples 80121, Italy*

*^2^Department of Materials, Environmental Sciences and Urban Planning, Polytechnic University of Marche, Ancona 60131, Italy*

*^3^ENEA - Agenzia per le Nuove Tecnologie, l'Energia e lo Sviluppo Economico Sostenibile, Via Anguillarese 301, 00123 Roma, Italy*

*^4^Department of Life and Environmental Sciences, Polytechnic University of Marche, Ancona 60131, Italy*

This file includes:

**Supplementary Table S1**

**Supplementary Figures S1 and S2**

**Supplementary Table S1.** **Output of the DISTLM analysis carried out to investigate the potential factors influencing the different taxa of the prokaryotic assemblage in the analysed sediments.** The following environmental and chemical variables were tested as potential drivers: sediment pH, sediment redox potential (Eh), the concentration of Biopolymeric carbon (BPC), phytopigments concentrations, grain size (% silt-clay), as well as the concentrations of HMs, aliphatic hydrocarbons (C>12) and total PAHs.

| *Bacterial taxa* | Driver | Adjusted R2 | SS | pseudo-F | P | Prop. (cum.%) |
| --- | --- | --- | --- | --- | --- | --- |
| Gammaproteobacteria *(dominant bacterial class)* | total PAHs | 4.86E-01 | 5.65E+03 | 9.46E+00 | 4.30E-03 | 48.6 |
|  | As | 8.03E-01 | 3.68E+03 | 1.44E+01 | 1.00E-04 | 80.3 |
|  | Cd | 8.99E-01 | 1.12E+03 | 7.59E+00 | 6.00E-04 | 89.9 |
| *Other abundant classes (each contributing more than 1% to the total reads count)* | grain size | 8.22E-01 | 5.30E+03 | 4.61E+01 | 1.00E-04 | 82.2 |
|  | Cu | 9.10E-01 | 5.71E+02 | 8.88E+00 | 2.00E-04 | 91.0 |
|  | Cr | 9.48E-01 | 2.44E+02 | 5.82E+00 | 9.00E-04 | 94.8 |
|  | total PAHs | 9.71E-01 | 1.46E+02 | 5.41E+00 | 8.50E-03 | 97.1 |
| *Rare classes (each contributing less than 1% to the total reads count)* | As | 7.53E-01 | 3.64E+03 | 3.05E+01 | 6.00E-04 | 75.3 |
|  | aliphatic hydrocarbons | 9.01E-01 | 7.16E+02 | 1.35E+01 | 1.00E-04 | 90.1 |
|  | Sediment Eh | 9.55E-01 | 2.60E+02 | 9.56E+00 | 6.00E-04 | 95.5 |


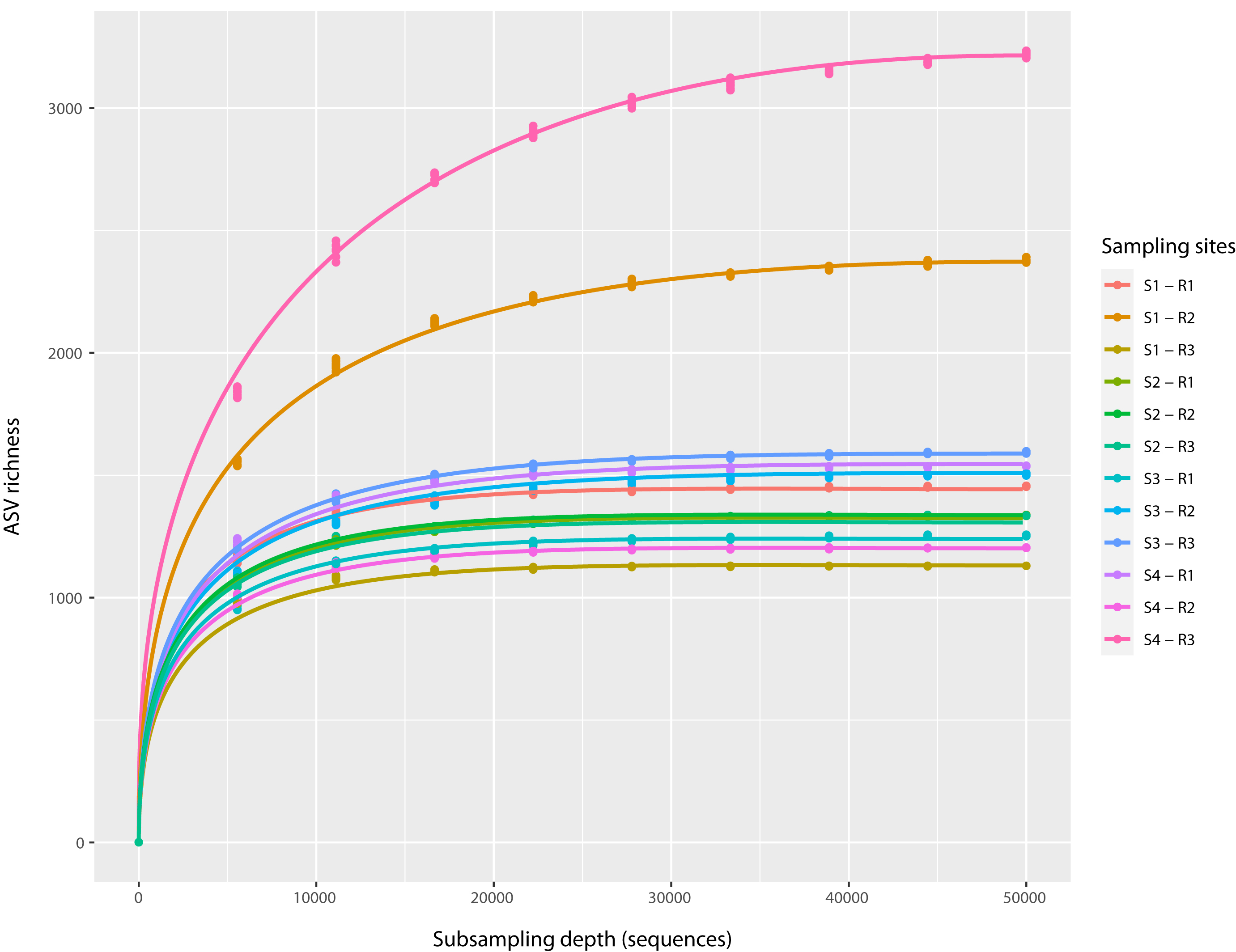


**Supplementary Figure S1.** **Rarefaction curve analysis showing the cumulative number of ASV detected for each sample to a maximum sequencing depth of 50000 sequences.**


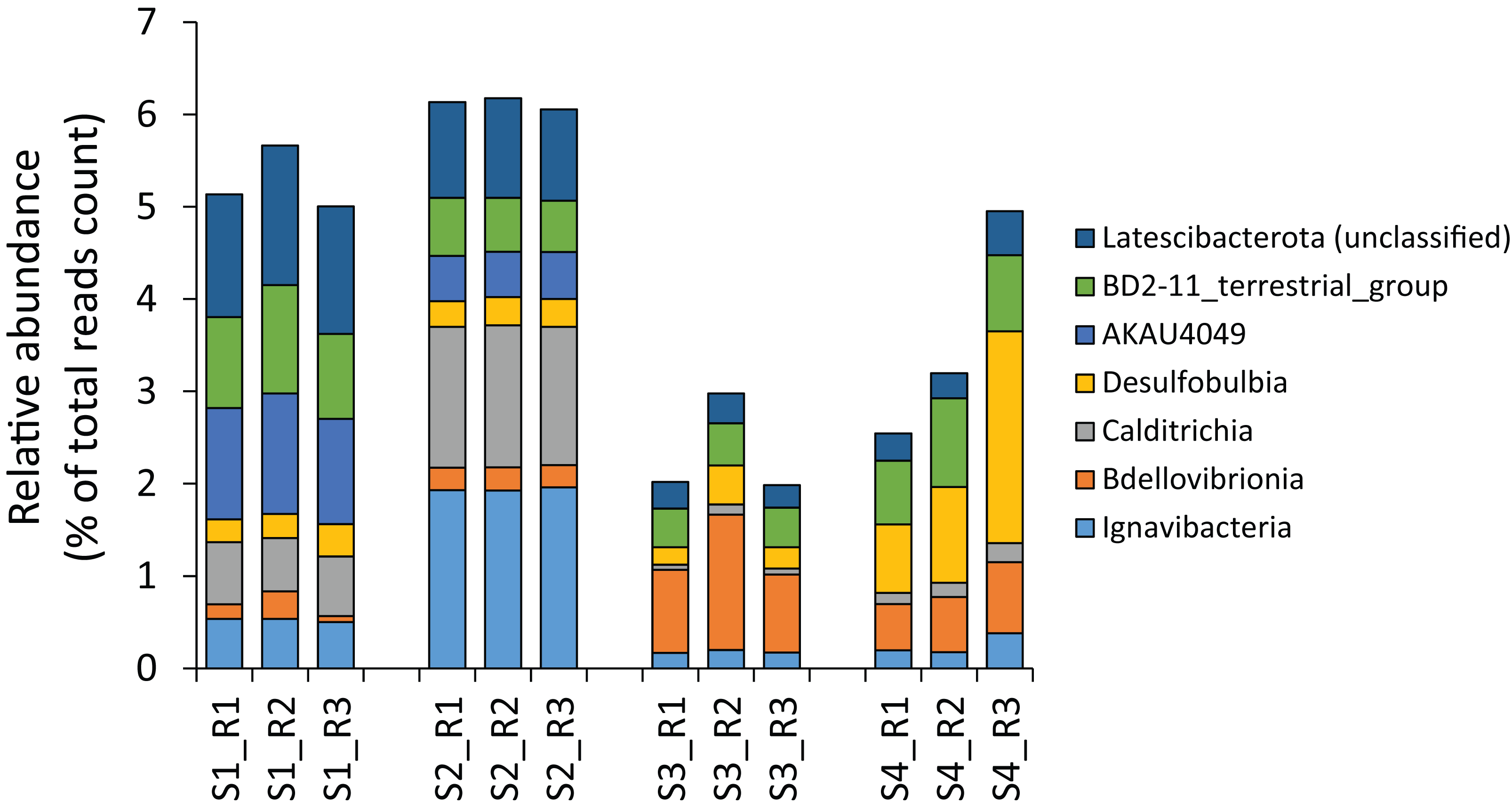


**Supplementary Figure S2. Highlight on the 7 bacterial classes that resulted rare within the dataset (i.e., on average contributing for less than 1% to the overall reads count across samples), but displayed peaks with relative contributions >1% in some samples.**
